# New insights into thyroid dysfunction in patients with inactivating parathyroid hormone/parathyroid hormone-related protein signalling disorder (the hormonal and ultrasound aspects): One-centre preliminary results

**DOI:** 10.3389/fendo.2022.1012658

**Published:** 2022-09-23

**Authors:** Dominika Januś, Dorota Roztoczyńska, Magdalena Janeczko, Jerzy B. Starzyk

**Affiliations:** ^1^ Department of Paediatric and Adolescent Endocrinology, Chair of Paediatrics, Institute of Paediatrics, Jagiellonian University Medical College, Krakow, Poland; ^2^ Department of Paediatric and Adolescent Endocrinology, University Children’s Hospital, Krakow, Poland; ^3^ Department of Genetics, Chair of Paediatrics, University Children’s Hospital, Krakow, Poland

**Keywords:** inactivating PTH/PTH-related protein signaling disorder, iPPSD, thyroid ultrasound, subclinical hypothyroidism, congenital hypothyroidism.

## Abstract

**Objective:**

This study aimed to present the spectrum of thyroid dysfunction, including hormonal and ultrasound aspects, in a cohort of paediatric and adult patients diagnosed with inactivating parathyroid hormone (PTH)/PTH-related protein signalling disorders 2 and 3 (iPPSD).

**Methods:**

The medical records of 31 patients from 14 families diagnosed with iPPSD between 1980 and 2021 in a single tertiary unit were retrospectively analysed. Biochemical, hormonal, molecular, and ultrasonographic parameters were assessed.

**Results:**

In total, 28 patients from 13 families were diagnosed with iPPSD2 (previously pseudohypoparathyroidism [PHP], PHP1A, and pseudo-PHP) at a mean age of 12.2 years (ranging from infancy to 48 years), and three patients from one family were diagnosed with iPPSD3 (PHP1B). Thyroid dysfunction was diagnosed in 21 of the 28 (75%) patients with iPPSD2. Neonatal screening detected congenital hypothyroidism (CH) in 4 of the 20 (20%) newborns. The spectrum of thyroid dysfunction included: CH, 3/21 (14.2%); CH and autoimmune thyroiditis with nodular goitre, 1/21 (4.8%); subclinical hypothyroidism, 10/21 (47.6%); subclinical hypothyroidism and nodular goitre, 1/21 (4.8%); primary hypothyroidism, 4/21 (19%); and autoimmune thyroiditis (Hashimoto and Graves’ disease), 2/21 (9.6%). Thyroid function was normal in 7 of the 28 (25%) patients with iPPSD2 and in all patients with iPPSD3. Ultrasound evaluation of the thyroid gland revealed markedly inhomogeneous echogenicity and structure in all patients with thyroid dysfunction. Goitre was found in three patients.

**Conclusion:**

The spectrum of thyroid dysfunction in iPPSD ranges from CH to autoimmune thyroiditis and nodular goitre. Ultrasonography of the thyroid gland may reveal an abnormal thyroid parenchyma.

## Introduction

Disorders of calcium phosphate metabolism related to parathyroid hormone (PTH) resistance and PTH signalling pathway impairment were first described by Fuller Albright et al. in 1942 and classified under the term pseudohypoparathyroidism (PHP) (1, 2). PHP results from the mutations and/or epigenetic alterations in the complex *GNAS* gene, encoding, among other transcripts, the G-stimulatory subunit (Gsα), located on chromosome 20q13.2 (3–5). These changes lead to loss of expression or function of Gsα, which impairs the transmission of stimulatory signals to adenylate cyclase with limited cyclic adenosine monophosphate (cAMP) generation (6). PHP is a rare genetic disorder with autosomal dominant transmission and parental imprinting, characterised by the target-organ unresponsiveness to hormones that share G protein-coupled receptors as the signalling pathway [PTH, thyroid-stimulating hormone (TSH), gonadotropins, glucagon, calcitonin, growth hormone–releasing hormone, neurotransmitters] (7, 8).

The main laboratory characteristics of PHP are hypocalcaemia and hyperphosphatemia associated with elevated PTH levels, which frequently encompass additional heterogeneous clinical features, referred to as Albright’s hereditary osteodystrophy (AHO) (9–14). In early infancy, the features may be subtle and extremely variable, ranging from early-onset rapid weight gain, subclinical hypothyroidism, round face, and ectopic subcutaneous ossifications to mild delay in acquisition of milestones (9–14). Other manifestations, such as brachymetacarpia, brachydactyly, brain calcifications, short stature, stocky build, progressively coarser facial features, and developmental delays, tend to develop relatively late in childhood (9–14).

Based on the 1980 classification of the cAMP response to PTH and Gsα functional activity, PHP was divided into PHP1A, pseudo-PHP (PPHP), and its variant progressive osseous heteroplasia (POH), PHP1B, PHP1C, and PHP-2 according to the presence or absence of AHO, together with an *in vivo* response to exogenous PTH and the measurement of Gsα protein activity in peripheral erythrocyte membranes *in vitro* (3, 4). PHP classification has become more complicated with recent molecular advancements; therefore, in 2016, the EuroPHP network developed a new classification that encompasses all disorders involving impairments in PTH and/or PTH-related protein cAMP-mediated pathways (1, 5). Inactivating PTH/PTH-related protein signalling disorder (iPPSD) is a new name for this group of disorders (1, 5). IPPSD is further subdivided, starting from the PTH receptor inactivating mutation as iPPSD1, inactivating *Gs*α mutations (PHP1A, PHP1C, PPHP, POH) as iPPSD2, loss of methylation of *GNAS* differentially methylated regions *DMRs* (PHP1B) as iPPSD3, *PRKAR1A* mutations as iPPSD4, *PDE4D* mutations as iPPSD5, and *PDE3A* mutations as iPPSD6. IPPSDx is reserved for unknown molecular defects and iPPSDn+1 for new molecular defects, which are yet to be described (1, 5).

IPPSD2 and iPPSD3 are the most frequent forms, and they will be presented in this study. The main feature of iPPSD2 and iPPSD3 is PTH resistance, and the second most frequent hormonal resistance is TSH resistance (1, 5).

The first description of hypothyroidism in patients with PHP was presented by Marx et al. in 1971 (15). This type of hypothyroidism has been characterised by elevated TSH levels, with normal or low normal (not exceeding the lower limit of the norm) concentrations of thyroid hormones, in the absence of goitre and antithyroid antibodies, a constellation termed subclinical hypothyroidism (15–18). In iPPSD2 resistance to TSH may be detected during neonatal screening, but most patients become clinically resistant to TSH during childhood or adolescence (19–22). In contrast, patients with iPPSD3 display TSH levels at the higher end of the normal range or mildly elevated levels (16). TSH resistance can also be present in patients with acrodysostosis due to pathogenic variants of *PRKAR1A* (iPPSD4) (17, 18).

There are few reports of thyroid ultrasound evaluation in iPPSD (7, 23–25). From a clinical paediatric endocrine standpoint, it is interesting and of practical value to determine how TSH resistance extrapolates to the ultrasound structure of the thyroid gland.

In this preliminary study, we investigated for the first time the ultrasound aspect of a wide spectrum of thyroid dysfunction in a cohort of patients diagnosed with iPPSD, with the aim of presenting the ultrasound characteristics of thyroid morphology in this disorder.

## Materials and methods

### Patients

This study included 31 patients (18 females and 13 males) with a mean age of 13.3 years and an age range from the neonatal period (in positive family history) to 48 years at the time of iPPSD diagnosis. The probants were recruited from 14 unrelated families. For clarity, each family was marked with an individual capital letter and a probant with the number 1.

According to the previous 1980 classifications, patients from families A to M were diagnosed with PHP1A and PPHP (patient M1) and from family N with PHP1B, based on clinical data that were molecularly confirmed in 11 families (**Table 1**). According to the EuroPHP 2016 classification, 13 families were diagnosed with iPPSD2 (known structural alterations in *GNAS* gene in 10 families, and although in three families the molecular study couldn’t be performed, the clinical manifestations are compatible with iPPSD2**)**. One family was diagnosed with iPPSD3 (**Table 1**).

**Table 1:** Clinical and molecular characteristics of patients and their families.

P	Year of birth	F/M	family h/o AHO	*GNAS* mutation/exon	Type of alteration	Transmission	AHO	1980 classification	2016 Euro PHP classification
A1	2007	F	-	ENST00000371085.8:c.[470_472delAAG];[=] ENSP00000360126.3:p.[[(p.Glu157del)];[(=)] exon 6	structural	*de novo*	+	PHP1A	iPPSD2
B1	1992	M	-	NA	-	*de novo*	+	PHP1A	iPPSD2 (clinical diagnosis)
C1	2009	M	C2 brother C3 mother	NA	-	maternal	+	PHP1A	iPPSD2 (clinical diagnosis)
D1	1992	F	-	ENST00000354359.11:c.[456_457insTG];[=] ENSP00000354359.11:p.[(Leu153CysfsTer21];[(=)] exon 6	structural	*de novo*	+	PHP1A	iPPSD2
E1	2005	M	E2 brother	ENST00000371085.8:c.[799delC];[=] ENSP00000360126.3:p.[(p.Leu 267CysfsTer5)];[(=)] exon 10	structural	maternal	+	PHP1A	iPPSD2
F1	2004	M	F2 brother F3 mother	ENST00000354359.12:c.[823A>T];[=] ENSP00000346328.7:[(p.Lys275Ter)];[=] exon 10	structural	maternal	+	PHP1A	iPPSD2
G1	2001	F	-	ENST00000354359.11:c.[c.772A>T];[=] ENSP00000346328.7:[(p.Ile258Phe) exon 10	structural	*de novo*	+	PHP1A	iPPSD2
H1	2002	M	H2 mother	ENST00000354359.11:c.[c.491A>C];[=] ENSP00000346328.7:p.[(Tyr164Ser];[(=)] exon 6	structural	maternal	+	PHP1A	iPPSD2
I1	1988	F	I2 son	ENST000000371085.8:c.[c.312+5G>A];[=] intron 4	structural	maternal	+	PHP1A	iPPSD2
J1	2012	F	-	ENST00000354359.11:c.[c.568_571 delGACT];[=] ENSP0000034632.8:[(p.Asp190MetfsTer14)];[(=)] exon 7	structural	*de novo*	+	PHP1A	iPPSD2
K1	1980	F	K2-mother of K1,K3-5 3 siblings of K1 K3- brother K4- brother K5- sister 3 daughters of K1 K6,K7, K8	ENST000000371085.8:c.[1009_1012delGCCAINSCCC];[=] ENSP00000360126.3:p.[(Ala337ProfsTer19)];[(=)] exon 12	structural	maternal	+	PHP1A	iPPSD2
L1	1993	F	-	NA	-	*de novo*	+	PHP1A	iPPSD2 (clinical diagnosis)
M1	2021	M	M2 father	ENST000000371085.8:c.[136_ 138dupCTG];[=] ENSP00000360126.3:p.[(p.Leu46dup)];[(=)] exon 1	structural	paternal	+	PPHP	iPPSD2
N1	1992	F	N2 mother N3 sister	LOM at A/B and 3.2-kb deletion at STX16 (STX16del) A/B	methylation	maternal	-	PHP1B	iPPSD3

A1–M2, diagnosed with iPPSD2; M1, diagnosed with a coding mutation on the paternal GNAS allele/pseudo-PHP/iPPSD2, and N1–N3, diagnosed with iPPSD3. LOM, loss of methylation; NA, non-available due to family problems; AHO, Albright`s Hereditary Osteodystrophy features; P-probants.

The 2016 classification was used in the present study.

In 57% (8 of the 14 families) of the families, there were other related iPPSD cases in the probant’s family (**Table 1**). In the largest family, iPPSD2 was diagnosed in members from three generations: grandmother (K2) and her four children (K1, K3, K4, K5) and in three daughters (K6, K7, K8) of mother K1.

### Methods

We performed a retrospective chart review of 31 patients (18 females) diagnosed with iPPSD between 1980 and 2021.

The analysis included the reasons for referral to the specialist by family doctor, and evaluation of calcium, phosphorus, PTH, and thyroid status (TSH, free thyroxine [fT4], thyroperoxidase antibody [TPOAb], thyroglobulin antibody [TGAb], TSH receptor antibody [TRAb]). All biochemical and hormonal assessments were routinely performed at the Department of Biochemistry at the University Children’s Hospital in Krakow, Poland, and were determined in a single fasting blood sample. PTH, TSH, and fT4 levels were measured using immunochemistry with an ADVIA Centaur machine, and TPOAb, TGAb and TRAb levels were assessed using the radioimmunoassay method with a Brams machine. All assessments were performed before inclusion in the study. *GNAS* gene molecular analysis was performed in 26 patients from 11 families prior to inclusion in the study in a commercial laboratory.

Thyroid ultrasound was performed for all patients at the time of thyroid dysfunction diagnosis. Thyroid ultrasound is routinely performed at our institution once a year in patients with thyroid dysfunction. The analysis included ultrasound features of the thyroid gland, elastography results (in two patients), and procedures [fine needle aspiration biopsy (FNAB) in two patients].

Ultrasonography (US) of the thyroid gland was performed at the University Children’s Hospital by ultrasound-certified paediatric endocrinologists with experience in paediatric US (DR > 35 years and DJ > 20 years of experience in paediatric US). Thyroid US was performed using a high-resolution Voluson 730 GE Medical System (8- to 12-MHz linear-array transducer) and a Samsung HS40 with elastography (LA3-16AD transducer). US was performed in the axial and longitudinal planes.

This study was approved by the relevant institutional review board (positive opinion number: 1072.6120.121.2022). Written informed consent was obtained from all the participants and/or their parents for the participation in the study and the use of ultrasound scans.

## Results

The clinical and molecular characteristics of the study group are presented in **Table 1**, and the clinical, hormonal, and biochemical data of the patients are presented in **Table 2**.

**Table 2:** Biochemical and hormonal characteristics of patients.

Patient number & gender	Endocrine assessment	age at PHP diagnosis[years]	age at thyroid dysfunction diagnosis [years]	calcium at diagnosis	phosphate at diagnosis	fT4 pmol/l, n: 10-25	TSH uIU/ml, n>2 yrs: 0.3-4.0, n<2 yrs: 0.4-9.1	fT4 pmol/l, n: 10-25	TPOAb, TGAb, TRAb	Thyroid ultrasound Volume (ml)echostructure	Newborn Screening[TSH]n<12 mIU/L
A1. F	5/52-congenital hypothyroidism 9 years-hypocalcaemia	9	5 weeks	Low	High	60.7	20.18	15.4	negative	0.9 [N] inhomogenous	I -4.68 II -17.9
B1. M	4 years- subclinical hypothyroidism 10 years- tonic- clonic seizures	10.8	4	Low	High	305.4; 248.5	9.2	10.1	negative	2.5 [N] inhomogenous	ND
C1. M	10/365-congenital hypothyroidism 4.1 years-hypocalcaemia	4.1	10 days	Low	High	243; 308.7	55.1	4.05	negative	0.8 [N] inhomogenous	27.8
C2. M	9/365-primary hypothyroidism, and hypocalcaemia	9 days	9 days	Low	High	310.9	20.5	5.5	negative	0.8 [N] inhomogenous	I -3.98 II-0.14
C3. F	35 years-subclinical hypothyroidism, and hypocalcaemia	35	35	Low	High	119	4.3	12.1	negative	9 [N] inhomogenous	ND
D1. F	11.2 years-seizure episodes without loss of consciousness 11.2 years- subclinical hypothyroidism	11.2	11.2	Low	High	965	5.3	10.8	negative	7.5 [N] inhomogenous	6.5
E1. M	3.7 years-subclinical hypothyroidism 13 yrs-nodular goiter. FNAB-Bethesda II 13.2 years-hypocalcaemia	13.2	3.7	Low	High	426.5	13.38	10.2	negative	10.1 [↑] inhomogenous,	7.66
E2. M	10/365 days-congenital hypothyroidism 10.7 years- autoimmune thyroiditis +nodular goiter. FNAB-Bethesda II 10.7 yrs-hypocalcaemia	10.7	10 days	Low	High	221.2	16.1	8.9	TPOAb: 579.9 U/l (+)	7.5 [↑] inhomogenous, pseudonodules, increased blood flow	13.11
F1. M	3/12-primary hypothyroidism 15.2 years- hypocalcaemia	15.2	3 months	Low	High	540.4; 498; 482	8.2	8.8	negative	0.9 [N] inhomogenous	6.06
F2. M	2/365-primary hypothyroidism 11.5 years-hypocalcaemia	11.5	2 days	Low	High	474.6	9.4	8.12	negative	0.8 [N] inhomogenous	1.13
F3. F	thyroid function normal	48	-	Normal	Normal	175	2.5	15.6	negative	10.5 [N] normal	ND
G1. F	15 years-subclinical hypothyroidism 18 years-diagnosed with iPPSD2 (distinctive AHO features)	18	15	Normal	Normal	74.1	7.5	10.2	negative	8.9 [N] inhomogenous	I -4.38 II-0.98
H1. M	14 years- primary hypothyroidism, and hypocalcaemia	14	14	Low	High	155.8	21.59	9.3	negative	9.4 [N] inhomogenous	2.87
H2. F	40 years-thyroid function normal, and hypocalcaemia	40	-	Low	High	91.1	3.5	14.5	negative	8.9 [N] normal	ND
I1. F	11 years- subclinical hypothyroidism 11 years-autoimmune thyroiditis 11 years- hypocalcaemia	11	11	Low	High	362.5; 524	7.84	11.8	TPOAb: 254.3 U/l (+)	7.4 [N] inhomogenous, pseudonodules, increased blood flow	6.7
I2. M	congenital hypothyroidism	since birth	2 days	Normal	Normal	83.8	16.77	11.8	negative	0.9 [N] inhomogenous	21.2
J1. F	subclinical hypothyroidism	1.1	1.1	Normal	Normal	119	20.79	10.6	negative	1.5 [N] inhomogenous	5.8
K1. F	3.2 years-sc ossifications and hypocalcaemia 15 years- Graves disease 16yrs- I recurrence, 17 yrs- II recurrence No remission 131J therapy at age 18.5 years	3.2	15	Low	High	154	0.09; 0.06; 0.0	49; 75; 77.6	TRAb: 50.7 U/l (+) TPOAb: 8936 U/l (+) TGAb: 125.3; 207.3 U/l (+)	17.7 [↑] inhomogenous, increased blood flow	ND
K2. F	diagnosed with PHP in adult endocrine clinic thyroid function normal	38	-	Low	High	164	2.5	15	negative	9.8 [N] normal	ND
K3. M	diagnosed with PHP in adult endocrine clinic thyroid function normal	20	-	Low	High	152	3.5	14.7	negative	9.1 [N] normal	ND
K4. M	diagnosed with PHP in infancy, died in infancy- agenesis of right lung & sepsis thyroid function normal	2 months	-	Low	High	164	5.6	15.1	negative	0.8 [N] normal	ND
K5. F	diagnosed with PHP in infancy, died in infancy-prematurity & sepsis thyroid function normal	1 month	-	Low	High	155	4.3	13.7	negative	0.91 [N] normal	ND
K6. F	4/365-subclinical hypothyroidism, and hypocalcaemia	4 days	4 days	Low	High	190	9.5	11.2	negative	0.8 [N] inhomogenous	5.8
K7. F	3/52-subclinical hypothyroidism, and hypocalcaemia	3 weeks	3 weeks	Low	High	221.9	12.1	10.3	negative	1.1 [N] inhomogenous	7.58
K8. F	10/365-subclinical hypothyroidism, and hypocalcaemia	10 days	10 days	Low	High	178	11.7	11.18	negative	1.2 [N] inhomogenous	7.7
L1. F	2.5 years-subclinical hypothyroidism 6 years- hypocalcaemia	6	2.5	Low	High	91.1; 143.9; 273	11.0	11.8	negative	3.8 [N] inhomogenous	7.9
M1. M	3/365-hypocalcaemia 3/12-subclinical hypothyroidism	3 days	3 months	Low	High	178.3	13.3	13.2	negative	0.9 [N] inhomogenous	*3.9*
M2. M	thyroid function normal 6 yrs- sc ossifications	6	-	Low	High	151.8	4.5	12.5	negative	2.75 [N] normal	4.5
N1. F	referred to endocrine unit after her mother was diagnosed with PHP1b thyroid function normal	8	-	Low	High	191.4	3.15	14.7	negative	5.1 [N] normal	6.7
N2. F	40 years -tonic seizures thyroid function normal	40	-	Low	High	157	1.5	15.2	negative	9.7 [N] normal	ND
N3. F	referred to endocrine unit after her mother was diagnosed with PHP1b thyroid function normal	19	-	Low	High	178	2.7	13.7	negative	8.6 [N] normal	ND

sc, subcutaneous; FNAB, fine needle aspiration biopsy; N, normal; I, first screening; II, second screening; ND, no data.

### Diagnosis

The reasons for referral to specialists by family doctor were as follows: 12, elevated TSH level (with low or normal fT4 level); 11, iPPSD already diagnosed and molecularly confirmed in family member(s); 4, abnormal newborn TSH screening results; 2, subcutaneous ossifications; and 2, hypocalcaemic seizures.

In 13 families, iPPSD2 was diagnosed based on PTH resistance and AHO manifestation, and in 10 of the 13 families, it was based on molecular evaluation of the *GNAS* gene (**Table 1**). Three families were lost to follow-up, and molecular assessments were not available, however the clinical manifestations are compatible with iPPSD2. Two infants with iPPSD2 died during infancy: K4, agenesis of the right lung and sepsis, and K5, prematurity and sepsis.

IPPSD2 was diagnosed at the mean age of 12.2 (range, 0.1–48) years.

In one family, the diagnosis of iPPSD3 was based on the lack of AHO manifestations together with PTH resistance (hypocalcaemia with increased serum PTH levels) and molecular confirmation (**Table 1**). IPPSD3 was first assessed in the mother in the adult endocrine centre and then in her two daughters.

In the whole cohort, hypocalcaemia and hyperphosphatemia were observed in 27 of the 31 patients and increased serum PTH levels in 31 of the 31 patients.

### Evaluation of thyroid function

The results are presented in **Table 2**.

Normal thyroid function with normal thyroid US and negative autoantibodies was found in seven patients with iPPSD2 and three patients with iPPSD3, respectively.

The mean TSH level in patients with normal thyroid function and morphology was 3.4 (range, 1.5–5.6 [normal value for age > 2 years, 0.3–4.0; normal value for age < 2 years, 0.4–9.1]) mIU/ml, the mean fT4 level was 14.5 (range, 12.5–15.6 [normal value, 10–25]) pmol/l, the mean thyroid volume was 6.6 (range, 0.8–10.5, within the normal range compared to Polish norms (26)) ml, and the mean age was 21.2 (range, 0.1–48) years.

Newborn screening results based on TSH assessment were available for 20 patients (the rest were born before the TSH screening programme was introduced in 1985). The mean TSH level was 8.1 (range, 0.14–27.8 [normal value, < 12]) mIU/l. Congenital hypothyroidism (CH) was diagnosed in 20% (4/20) of the newborns screened.

Thyroid dysfunction was diagnosed in 21 of the 28 (75%) patients with iPPSD2. The spectrum of thyroid dysfunction included: CH, 3/21 (14.2%); CH with development of autoimmune thyroiditis and nodular goitre, 1/21 (4.8%); subclinical hypothyroidism, 10/21 (47.6%); subclinical hypothyroidism and nodular goitre, 1/21 (4.8%); primary hypothyroidism, 4/21 (19%); and autoimmune thyroiditis (Hashimoto and Graves’ disease), 2/21 (9.6%).

The mean TSH level in patients with hypothyroidism was 14.7 (range, 4.3–55.1) mIU/ml, the mean fT4 level was 10.26 (range, 4.05–15.4) pmol/l, the mean thyroid volume was 3.3 (range, 0.8–9.4) ml, and the mean age was 5.3 (range, 0.1–35) years.

The median TSH level in three patients with goitre was 9.8 (range, 0.09–16.1) mIU/ml, the median fT4 level was 22.7 (range, 8.9–49) pmol/l, the median thyroid volume was 11.7 (range, 7.5–17.7), ml, and the median age was 9.8 (range, 3.7–15) years.

Autoimmune thyroiditis was diagnosed in three patients: one with CH (E2), one with subclinical hypothyroidism (E1), and one (K1) with overt hyperthyroidism (Graves’ disease). Patient K1 was diagnosed with thyrotoxicosis at the age of 15 years (TSH, 0.09 uIU/ml; fT4, 49 pmol/l with positive TPOAb and TRAb). Thyroid scintigraphy in this patient revealed a homogenous increased intake of Tc-99m (3.4 mCi), and the thyroid gland was enlarged asymmetrically (mainly in the right lobe). An antithyroid drug (thiamazole) was introduced along with calcitriol and calcium carbonate. After two recurrences and inability to achieve long-term remission, radioiodine therapy was offered to the patient, who remained clinically and biochemically euthyroid on levothyroxine after radioiodine ablation.

The mean age at iPPSD2 diagnosis was 12.2 (range, 0.1–48) years, and the mean age at diagnosis of thyroid dysfunction was 5.4 (range, 0.1–35) years (**Table 2**). In 9 of the 21 (42.9%) patients, thyroid dysfunction was diagnosed before iPPSD2 confirmation, and the mean time difference between these two diagnoses was 8.1 (the longest 14.9 years) years. In two (9.5%) patients, iPPSD was diagnosed prior to and in 10 patients (47.6%) at the same time as thyroid dysfunction.

There was no evidence of other endocrine problems in these patients, except for PTH resistance. The onset of puberty was within the normal age range, as was the menstrual cycle in female patients. Normal fertility was observed in seven families (**Table 1**).

The patients received therapy as required to achieve normocalcaemia and euthyroidism (calcium carbonate, calcitriol (when alfacalcidol was unavailable) or alfacalcidol, levothyroxine).

### Ultrasound study

The thyroid ultrasound findings in the study patients varied from normal thyroid structure to nodular goitre. Thyroid ultrasound was normal in patients with iPPSD3 and in seven patients with iPPSD2 with normal thyroid function.

In patients with autoimmune thyroiditis and hypothyroidism, a typical image of ‘pseudonodules’ (hypoechogenic areas < 3 mm) with increased vascularity was observed.

In a patient with Graves’ disease, US of the thyroid gland (scans are not shown due to poor quality) revealed a hypoechogenic and inhomogeneous goitre, with a thyroid volume of 17.7 (normal value, ≤ 15) ml and increased blood flow.

**Figure 1:**
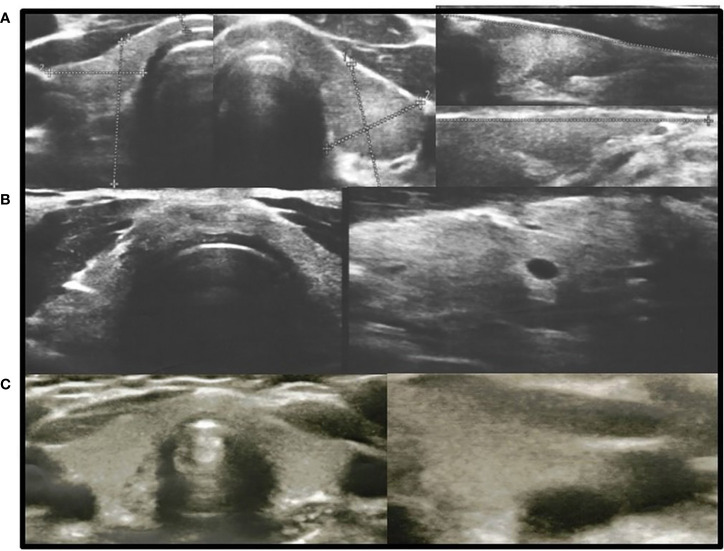
Axial and longitudinal ultrasonography scans of the thyroid gland in three patients with hypothyroidism presenting with decreased echogenicity and irregular thyroid structure. Blood flow is normal. **(A)**, Patient H1; **(B)**, Patient D1; **(C)**, Patient M1. Samsung HS40.

**Figure 2:**
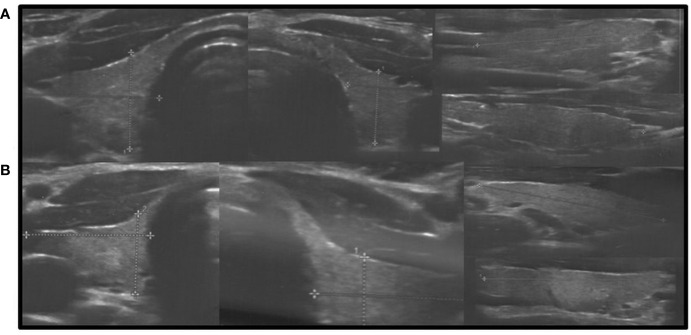
Axial and longitudinal ultrasonography scans of the thyroid gland in two patients presenting with decreased echogenicity and irregular coarse/nodular thyroid structure. Blood flow is normal. **(A)**, Patient G1; **(B)**, Patient L1. Samsung HS40.

**Figure 3:**
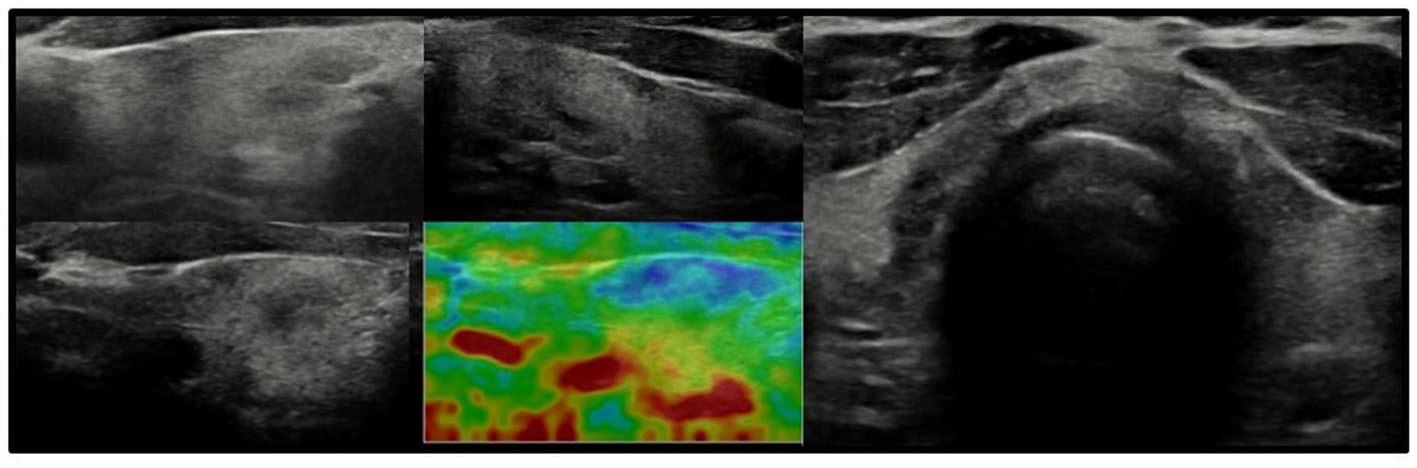
Axial and longitudinal ultrasonography scans of the thyroid gland in patient F1 presenting with irregular echostructure and echogenicity of the thyroid gland. Elastography reveals that the hyperechogenic areas are stiffer than the hypoechogenic areas of the thyroid gland. Blood flow is normal. Samsung HS40.

**Figure 4:**
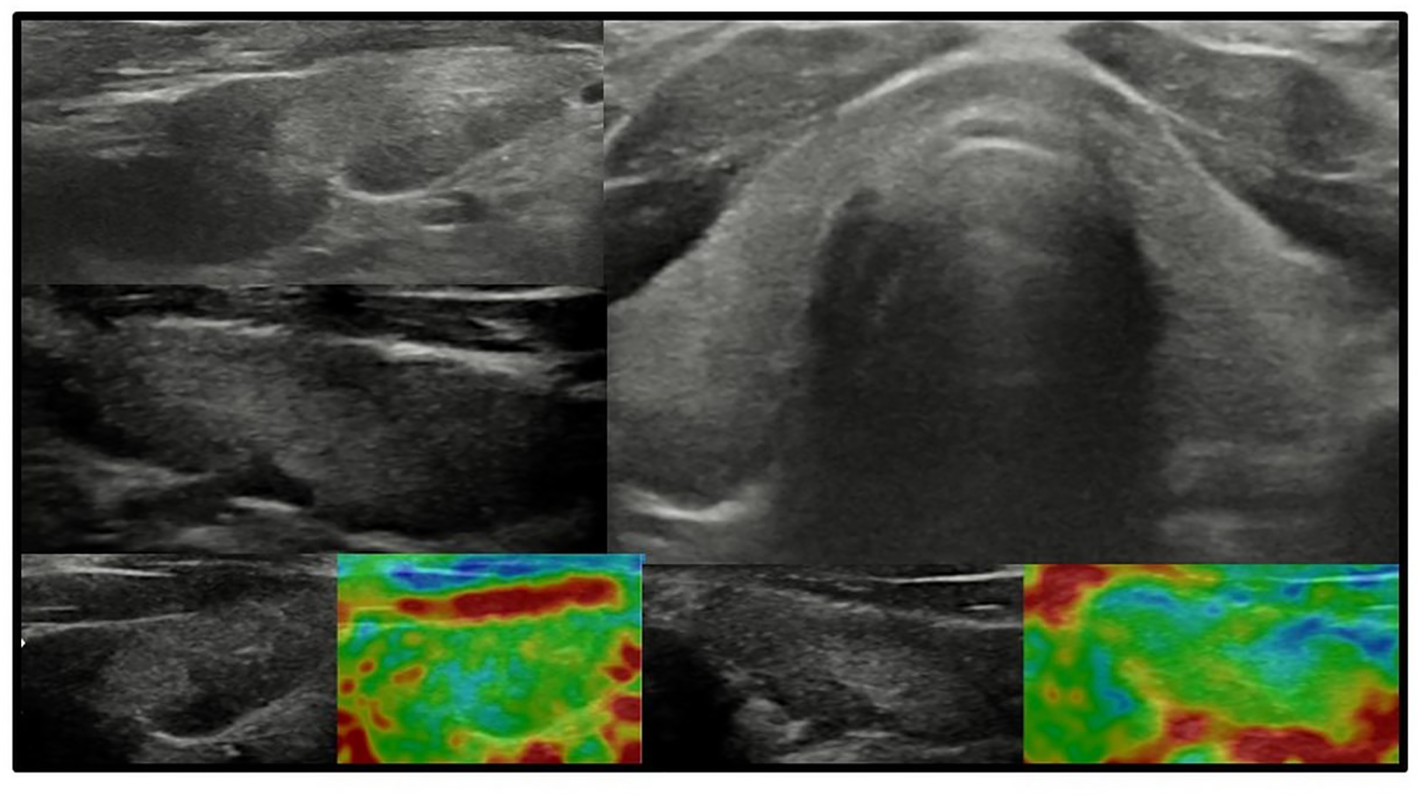
Axial and longitudinal ultrasonography scans of the thyroid gland in patient F2 presenting with irregular echostructure and echogenicity of the thyroid gland. Elastography reveals that the hyperechogenic areas are stiffer than the peripheral areas of the thyroid gland. Blood flow is normal. Samsung HS40.

In all other patients with thyroid dysfunction (congenital, subclinical, and primary hypothyroidism), ultrasound scans revealed a highly inhomogeneous thyroid parenchyma (**Figures 1**–**4**). Elastography performed in two patients revealed heterogenicity in the elasticity of the thyroid gland, with hyperechogenic areas presenting higher stiffness (**Figures 3**, **4**). Blood flow was normal.

The thyroid gland volumes were within the normal ranges, except for three patients with goitre (K1, E1, E2) (**Table 1**). Nodular goitre was found in two patients (brothers E1 and E2). The nodules were less than 1 cm: 4 × 6 mm in the left lobe in E1 and 6 × 5 mm in the right lobe in E2, with irregular echogenicity and normal blood flow. FNAB results were classified as Bethesda II (benign lesion).

## Discussion

TSH resistance can cause a spectrum of thyroid dysfunction, from compensated to overt hypothyroidism (27–29), and may be diagnosed before the appearance of PTH resistance in iPPSD2 (15, 27–35). The mechanisms of the gradual development of PTH resistance are, for the time being, hypothesized, possibly related to the type of genetic alteration in *GNAS* gene (30–32).

In the present study, thyroid dysfunction was diagnosed before iPPSD2 confirmation in less than half of the patients (42.9%); however, the mean time difference between these two diagnoses was 8.1 years, with the longest being almost 15 years. This is less than that in other studies due to the many related familiar cases in our cohort, enabling earlier iPPSD diagnosis (14). Nevertheless, it is concerning that establishing iPPSD diagnosis is considered difficult, although AHO features were evident in all patients. On the other hand, as presented by Elli et al., performing an early diagnosis is challenging, due to the clinical overlap among iPPSD subtypes and the variable presentation and severity of signs and symptoms, that have led to the misdiagnosis of some of the patients without molecular confirmation (19). We are aware that three patients in this study, without molecular confirmation, are not exactly iPPSD2 patients, though the clinical manifestations were compatible with iPPSD2. The identification of the underlying genetic or epigenetic defect is important to perform a diagnosis, allowing an appropriate genetic counseling, treatment, screening for complications and follow-up (19).

Evidence of TSH resistance is often present at birth, and some patients may be diagnosed through neonatal screening with CH (35–39).

It is useful for clinical practitioners to recall the classification of CH when assessing newborn screening programme results. CH is divided into central, primary, and peripheral hypothyroidism (27). The most common primary CH is further divided into thyroid dysgenesis, thyroid dyshormonogenesis, and TSH resistance, including two main subtypes: defects in the TSH receptor and abnormalities in the G-protein α-subunit (27). Therefore, in certain situations of excessive weight increase with therapeutic levothyroxine or if there are more cases of CH in one family (as presented in this study), infants with ‘CH’ should also be screened for potential iPPSD (14, 28, 30).

The blood spot TSH concentrations usually do not reach the cut-off values for recall (in our country, it is > 12 mIU/l) (14, 20). Four patients in this study were detected by systematic postnatal CH screening based on TSH evaluation (TSH, 17.9, 27.8, 13.1, and 21.2 mIU/l). Interestingly, lowering the TSH cut-off value to > 6 would increase the detection of hypothyroidism from 20% (4/20) to 60% (12/20) of the newborns with iPPSD2 in our study, as proposed by Langham et al. (40). All children who were not detected by screening were diagnosed with hypothyroidism later and were subsequently treated with levothyroxine. Whether earlier therapy would have a positive effect on their development remains unclear. According to some studies, despite a prompt diagnosis of hypothyroidism after birth and initiation of treatment, this does not seem to prevent motor or cognitive delay (41).

Most patients become clinically resistant to TSH during childhood or adolescence (19–21). In the patients with hypothyroidism with iPPSD2 in this study, the average TSH level was 14.7 mUI/l. Patients with more severe TSH resistance presented with thyroid function tests consistent with primary hypothyroidism; however, the TSH level was < 56 mIU/l. At the time of our study, all patients with iPPSD3 had normal thyroid function. These findings are similar to those reported by other studies (14, 28–30, 36, 42).

There are only a few detailed reports on the coincidence of autoimmune thyroiditis and iPPSD (23, 24, 43–46). The coincidence of Graves’ disease (with positive TRAb) and clinically diagnosed iPPSD2 was described by Uchimura et al. and iPPSD3 by Gerhardt et al. (23, 46). Mukherjee et al. described a case of iPPSD that subsequently developed multiple autoimmune disorders, such as celiac disease, hypothyroidism, and type 1 diabetes (43). Zeng et al. reported a patient with Turner syndrome who simultaneously had iPPSD and autoimmune thyroiditis (24). Krysiak et al. described a case of iPPSD with concurrent chronic thyroiditis and pernicious anaemia (44).

Thyroid autoantibodies are usually not observed in patients with iPPSD2 and iPPSD3 (36). Nevertheless, given the high prevalence of autoimmune thyroid disease, the presence of thyroid autoantibodies does not exclude TSH resistance (36). It is not yet known whether this association is purely coincidental, and additive or whether there are some common underlying pathophysiological mechanisms and whether they might be causally related, but it requires further studies (43, 47).

Although the prevalence of hypothyroidism (subclinical, congenital, or not) has been extensively investigated in patients with iPPSD, precise thyroid ultrasound findings in these disorders have not been reported.

Ultrasound evaluation of the thyroid glands in patients with iPPSD revealed more diverse thyroid images than those previously suspected from normal thyroid US *via* a picture of thyroiditis to nodular goitre. Thyroid ultrasound images of the study patients with autoimmune thyroiditis revealed typical pseudonodules, diffuse thyroiditis and increased blood flow. Analysis of thyroid ultrasound in patients with subclinical, primary, and congenital hypothyroidisms revealed marked inhomogeneity in thyroid structure and echogenicity. The images in the latter group of patients differ from those observed in the most common cause of thyroid dysfunction in children, such as autoimmune thyroiditis, as presented in our previous studies (48, 49). In autoimmune thyroiditis in children, lymphocytic infiltration can be observed as diffuse thyroiditis, irregular thyroiditis, pseudonodules, hypoechogenic thyroid, or nodular form, and the blood flow may be increased in the hypoechogenic areas in the thyroid parenchyma, as previously presented (48, 49). In the present study TSH resistance was observed rather as inhomogeneity, and the blood flow was not increased in the hypoechogenic areas, but it requires further studies. Elastography revealed that the hyperechogenic areas were stiffer than the hypoechogenic areas in the thyroid gland.

We are aware that in our preliminary study we could not present typical ultrasound features for TSH resistance, as we were not able to exclude other causes of hypothyroidism, such as autoimmune thyroiditis without circulating antibodies (up to 20% of the thyroiditis, by FNAB) and an iodine deficient status. However, we suggest that in these particular patients, especially with unexplained hypothyroidism, showing parenchymal changes at US, with no increased blood flow, other causes of concomitant primary hypothyroidisms should be excluded.

Studies on thyroid imaging in iPPSD are insufficient (7, 23–25). Elli et al. found normal thyroid scans in a cohort of patients (7). Gerhard et al. found a normal-sized thyroid in a patient with iPPSD and Graves’ disease; however, echogenicity and echostructure were not typical for Graves’ disease (23). Zeng et al. reported heterogeneous hypoechoic changes involving both thyroid lobes (likely pseudonodules typical of thyroiditis) (24). Lu et al. reported an atrophic thyroid gland in a patient with iPPSD (25).

The majority of children we described had normal-sized thyroid glands on ultrasound imaging, although TSH levels were above the upper normal range. These findings are suggestive of

failure of the thyroid gland to respond appropriately to TSH and are compatible with the presence of a defect in the TSH receptor-adenylate cyclase complex (20). However, the presence of a goitre in three patients may indicate that this defect is caused by a partial deficiency (approximately 50% reduction) of the α-subunit of the stimulatory G protein (Gsα) (27). Resistance to TSH is generally mild, which may be explained by partial imprinting in the thyroid with incomplete silencing of the paternal allele (27). Whether this explains the highly heterogeneous morphology of the thyroid gland is unknown but possible because TSH stimulates the growth and function of thyroid follicular cells (27).

Our retrospective, preliminary clinical study has some limitations. This was a retrospective analysis of patients referred to a single tertiary paediatric centre. Our study group was significantly small to determine prevalence; however, we were able to present novel ultrasound insights into thyroid gland dysfunction in iPPSD, which may be helpful in iPPSD diagnosis.

## Summary

The spectrum of thyroid dysfunction in iPPSD is broad, ranging from CH to autoimmune thyroiditis and nodular goitre. US of the thyroid gland may reveal an abnormal thyroid parenchyma.

## Data availability statement

The raw data supporting the conclusions of this article will be made available by the authors, without undue reservation.

## Ethics statement

This study was approved by the relevant institutional review board (positive opinion number: 1072.6120.121.2022). Written informed consent was obtained from all the participants and/or their parents. Written informed consent to participate in this study was provided by the participants’ legal guardian/next of kin.

Written informed consent was obtained from the individual(s), and minor(s)’ legal guardian/next of kin, for the publication of any potentially identifiable images or data included in this article.

## Author contributions

Study design: DJ and DR. Study conduct: DJ, DR, and MJ. Data collection: DJ and DR. Data analysis: DJ and DR. Data interpretation: DJ and DR. Drafting manuscript: DJ and DR. Revising manuscript content: DJ, DR, MJ, and JS. Approving final version of manuscript: DJ, DR, MJ, and JS. DJ takes responsibility for the integrity of the data analysis. All authors contributed to the article and approved the submitted version.

## Acknowledgments

We would like to thank Editage (www.editage.com) for English language editing.

## Conflict of interest

The authors declare that the research was conducted in the absence of any commercial or financial relationships that could be construed as a potential conflict of interest.

## Publisher’s note

All claims expressed in this article are solely those of the authors and do not necessarily represent those of their affiliated organizations, or those of the publisher, the editors and the reviewers. Any product that may be evaluated in this article, or claim that may be made by its manufacturer, is not guaranteed or endorsed by the publisher.
